# Targeted Clearance of Senescent Endothelium by GPNMB‐Specific CAR‐T Cells

**DOI:** 10.1155/jimr/1976386

**Published:** 2026-08-05

**Authors:** Peijie Yu, Jie Du, Yan Liu, Mei Zhou

**Affiliations:** ^1^ Beijing Anzhen Hospital, Capital Medical University, Beijing Institute of Heart Lung and Blood Vessel Diseases, Beijing 100029, China, ccmu.edu.cn

**Keywords:** CAR-T cells, endothelial cells, GPNMB, Matrigel, senescence

## Abstract

Endothelial cells (ECs) serve as crucial components of blood vessels and are vital for preserving vascular health in humans. The aging of these cells significantly accelerates vascular aging. Factors released by aging ECs serve as key initiators of arterial dysfunction and various cardiometabolic diseases. As a result, the targeted removal of aging ECs from injured tissues could reduce these issues and potentially improve lifespan. In this research, we explored the therapeutic capabilities of chimeric antigen receptor (CAR)‐T cells designed to focus on senescent cells as potential senolytic agents. We identified GPNMB, a transmembrane glycoprotein, as a protein characterized by extensive expression during cellular senescence. Our findings demonstrate that CAR‐T cells specifically targeting GPNMB can efficiently remove senescent cells in vitro. In addition to this, the engineered CAR‐T cells significantly clear replicative and doxorubicin (Dox)‐induced senescent human umbilical vein ECs (HUVECs). Notably, these CAR‐T cells successfully eradicated senescent HUVECs within a Matrigel matrix implanted subcutaneously in mice, thereby creating a simulated in vivo environment that mimics physiological conditions. The findings emphasize the possibilities of CAR‐T cells in treating EC senescence.

## 1. Introduction

The process of aging in organisms is intricately linked to the dysfunction of multiple organs. The aging of organs is not a uniform occurrence; rather, it is a variable and dynamic phenomenon, with the vascular system standing out as one of the first and most significantly impacted areas [1]. Vascular aging plays a crucial role in initiating systemic aging by interacting extensively with multiple organs and diverse circulating factors. Thus, intervening to slow down vascular senescence could be a promising approach to address diseases associated with aging.

The introduction of a chimeric antigen receptor (CAR)‐encoding vector enables T cells to recognize specific antigens on target cells, thereby granting them the ability to precisely target and destroy these cells. CAR‐T cell therapy was first implemented in patients suffering from chronic lymphocytic leukemia, resulting in long‐lasting remission [2]. Subsequently, favorable results were observed in B cell non‐Hodgkin lymphoma, acute lymphoblastic leukemia (ALL), autoimmune disorders, and cardiac fibrosis [3–7]. Recent advancements in the field of immunotherapy have highlighted the promising role of CAR‐T cells in tackling cellular senescence, particularly in models of rheumatoid arthritis (RA) and liver fibrosis (LF) [8]. As of now, CAR‐T cell therapy represents a promising strategy for treating age‐related diseases.

GPNMB is recognized as a type I transmembrane protein, first discovered in melanoma cell lines [9]. This protein has various functions in the aging process. Transcriptomic analysis of senescent vascular endothelial cells (ECs) has indicated a specific increase in GPNMB, highlighting its potential role as a senescence‐specific antigen [10]. Therefore, the removal of GPNMB‐positive senescent ECs could be beneficial in alleviating pathological vascular aging. To support clinical applications, we developed a vector that encodes a single‐chain variable fragment (scFv) originating from a human GPNMB antibody aimed at targeting senescent ECs.

## 2. Materials and Methods

### 2.1. Cell Culture and Senescence Induction

Human umbilical vein ECs (HUVECs) were cultured in the EC medium (ECM, ScienCell, Carlsbad, CA, USA) supplemented with 5% fetal bovine serum (FBS), EC growth supplement (ECGS), and 1% penicillin/streptomycin at 37°C in a humidified atmosphere containing 5% CO_2_.

Replicative senescence of HUVECs was induced by subculturing. Cells at passage 5 (P5) and passage 30 (P30) served as young and senescent cells, respectively. We used doxorubicin (Dox) to induce premature senescence. Young HUVECs were treated with 10 nM Dox (Sigma–Aldrich, St. Louis, MO, USA) for 48 h, and then, the medium was replaced with complete medium and further incubated for 48 h [11–14].

### 2.2. Senescence‐Associated β‐Galactosidase (SA‐β‐Gal) Assay

SA‐β‐Gal activity in senescent HUVECs was detected using a SA‐β‐Gal staining kit (Abcam, Cambridge, UK) according to the manufacturer’s recommendations. Cells were fixed in a fixative solution for 10–15 min at room temperature (RT), followed by two washes with phosphate‐buffered saline (PBS). Subsequently, cells were incubated in SA‐β‐gal staining solution overnight at 37°C without CO_2_. The cells were then observed under a microscope for the development of a blue color.

### 2.3. qRT‐PCR Analysis

Total RNA was obtained from cells using the TRIzol reagent (Thermo Fisher). The RevertAid First Strand cDNA Synthesis Kit (Thermo Fisher) was employed for carrying out reverse transcription reactions. SYBR Green PCR Master Mix (Vazyme, Nanjing, Jiangsu, China) was utilized for qPCR in a CFX‐connected real‐time system (Bio‐Rad, Hercules, CA, USA). The primer sequences used for quantitative PCR were as follows (5′–3′): GPNMB: F‐AAGATTGCCACTTGATGCCG, R‐TCCCTCATGTAAGCAGAAGGTC; P16 ^INK4A^: F‐ATGGAGCCTTCGGCTGACT, R‐GTAACTATTCGGTGCGTTGGG; GAPDH: F‐AATGCATCCTGCACCACC, R‐ATGCCAGTGAGCTTCCCG.

### 2.4. Western Blotting

Cells were washed twice with PBS and lysed on ice for 30 min in lysis buffer containing the protease inhibitor cocktail (Thermo Fisher Scientific), followed by quantification of the protein extracts. Subsequently, the lysates were resolved using SDS–polyacrylamide gel electrophoresis, and proteins were transferred to PVDF membranes (Millipore). The membranes were then blocked with 5% skimmed milk for 1 h and incubated at 4°C overnight, shaking gently with the anti‐GPNMB (#13251, Cell Signaling Technology), anti‐p16^INK4A^ (ab108349, Abcam), and anti‐GAPDH (ab8245, Abcam) primary antibodies. Following three washes with tris‐buffered saline‐Tween (TBST), the membranes were then exposed to infrared fluorescence‐labeled secondary antibodies (Li‐Cor Bioscience, Lincoln, NE, USA) at RT for 1 h. The membranes were scanned by the Odyssey infrared imaging system (Li‐Cor Bioscience).

### 2.5. GPNMB‐CAR Design and Construction

The anti‐GPNMB scFv used in this study was derived from a previously reported anti‐GPNMB monoclonal antibody (CR011), originally described by Tse et al. [15] and further developed in subsequent studies targeting GPNMB‐expressing tumors. The cDNA sequence of the scFv fragment of a human GPNMB monoclonal antibody was synthesized and cloned into a CAR‐P2A‐EGFP lentiviral transfer vector (Genechem, Shanghai, China) at the BamHI restriction site. The vector contained a CD8α hinge and transmembrane region, a CD137 (4–1BB) costimulatory domain, and a CD3ζ intracellular signaling domain. The GPNMB‐CAR lentivirus was packaged using 293T cells by Genechem. The vector lacking the GPNMB scFv was used as the control vector. The sequences for CD8α hinge and transmembrane region, CD137 costimulatory domain, and CD3ζ intracellular signaling domain are provided in Supporting Information 1: Table S1.

### 2.6. T Cell Transduction and Expansion

Peripheral blood mononuclear cells (PBMCs) were isolated from the whole blood of healthy volunteers (age 25–40 years; 3 males and 3 females; Chinese Han ethnicity) through density gradient centrifugation utilizing Ficoll‐Paque PLUS (Amersham Biosciences). Written informed consents were obtained from all donors. This study was approved by the Ethics Committee of the Beijing Anzhen Hospital, Capital Medical University. T cells from humans were exclusively isolated with the EasySep Direct Human T Cell Isolation Kit (StemCell). The cells were cultured in advanced RPMI 1640 medium (Thermo Fisher Scientific) containing 10% heat‐inactivated FBS at a density of 10^6^ per mL, activated by CD3/CD28 T cell activator (25 μL/mL, Life Technologies), and expanded with 100 U/mL hIL‐2, 10 ng/mL hIL‐7, and 5 ng/mL hIL‐15 (PeproTech). After 24 h, the activated T cells were transduced with control or GPNMB‐CAR‐expressing lentiviral particles at an MOI of 10 using spinoculation. The culture plates were spun at 2500 rpm for 90 min at 32°C. The cells were cultured at 37°C in a humidified incubator with 5% CO_2_. The complete medium with cytokines was supplied every other day.

### 2.7. Cytotoxicity Assays

Senescent and control HUVECs were plated in 96‐well plates at a density of 10^4^ cells per well. On the subsequent day, effector T cells were added to each well at various effector‐to‐target (E:T) ratios (1:1, 2.5:1, and 5:1) to assess their cytotoxic capabilities against the target cells. The morphologic changes of target cells after coculture were observed using a Leica microscope. Supernatant samples were collected at 6, 12, and 24 h after coculture. Lactate dehydrogenase (LDH) released by injured target cells was determined using the LDH Cytotoxicity Assay Kit (J2380, Promega) according to the manufacturer’s instructions. Specific lysis was calculated as ([experimental LDH release − medium background]/[maximum LDH release control− medium background]) × 100%. The maximum LDH release control was obtained by lysing target cells with 10% Triton X‐100 for 20 min prior to supernatant collection. LDH release in target cells cultured without T cells served as a medium background. Furthermore, the concentrations of human interferon‐gamma (IFN‐γ) in the supernatant at 24 h were measured using the Human IFN‐γ CBA Flex Set (BD Biosciences) according to the manufacturer’s protocols.

### 2.8. Flow Cytometry

For GPNMB surface expression in HUVECs, the cells were incubated with biotin anti‐CD31 antibody and anti‐GPNMB antibody at RT for 30 min, avoiding light. After washing with PBS, FITC‐conjugated streptavidin antibody was added to the cells and stayed for an additional 30 min avoiding light. T cells were harvested, washed twice with PBS, and resuspended in 100 μL PBS. For CAR detection, the cells were incubated with the His‐tagged recombinant human GPNMB extracellular domain at RT for 30 min. After washing with PBS, a fluorescence‐conjugated anti‐His antibody was added to the cells and stayed for an additional 30 min avoiding light. For T cell activation detection, the cells were incubated with a fluorescence‐conjugated anti‐CD69 antibody at RT for 30 min, avoiding light. After washing twice with PBS, data acquisition was performed using an LSR Fortessa cell analyzer (BD Biosciences) and analyzed with FlowJo software (BD, Version 10.8.1). The complete gating strategy is provided in Supporting Information 3: Figures S3–S5. All antibody information has been provided in Supporting Information 2: Table S2.

### 2.9. Matrigel Plug Assay

Matrigel Matrix (Corning) was diluted to 8 mg/mL in PBS on ice. 10^6^ of senescent or control HUVECs with or without an equal number of CAR‐T cells were mixed in 200 μL of the Matrigel on ice. The solution was then subcutaneously injected into the abdominal midline of the anesthetized 8‐week‐old male NSG mice (Viewsolid Biotech, Beijing, China) using a 1 mL prechilled syringe. After 3 days, the Matrigel plugs were excised and fixed with 4% paraformaldehyde. After embedding with Tissue‐Tek optimum cutting temperature (OCT) compound, the Matrigel plugs were sectioned and stained with anti‐human CD31 antibody (Servicebio, Wuhan, Hubei, China).

### 2.10. Immunofluorescence Staining

The cells were fixed with 4% paraformaldehyde for 15 min, washed twice with PBS with 3 min intervals, and then permeabilized with 0.03% Triton X‐100 for 5 min. Following blocking with 10% goat serum for 20 min at RT, and incubation with anti‐GPNMB antibody (1:100, Cell Signaling Technology) and anti‐CD31 antibody (1:100, Servicebio) at 4°C overnight, followed by incubation with the fluorescence‐labeled secondary antibody (1:500) for 1 h at RT. Nuclei were counter stained with DAPI (Abcam). Fluorescent signals were captured under a laser confocal microscope (Leica, USA).

### 2.11. Statistics

Statistical significance was assessed using GraphPad Prism 9 software (La Jolla, CA). Data represent the mean ± SD. An unpaired Student’s *t*‐test was utilized for comparing the two groups.

## 3. Results

### 3.1. Upregulation of GPNMB Expression in Senescent HUVECs

**Figure 1:**
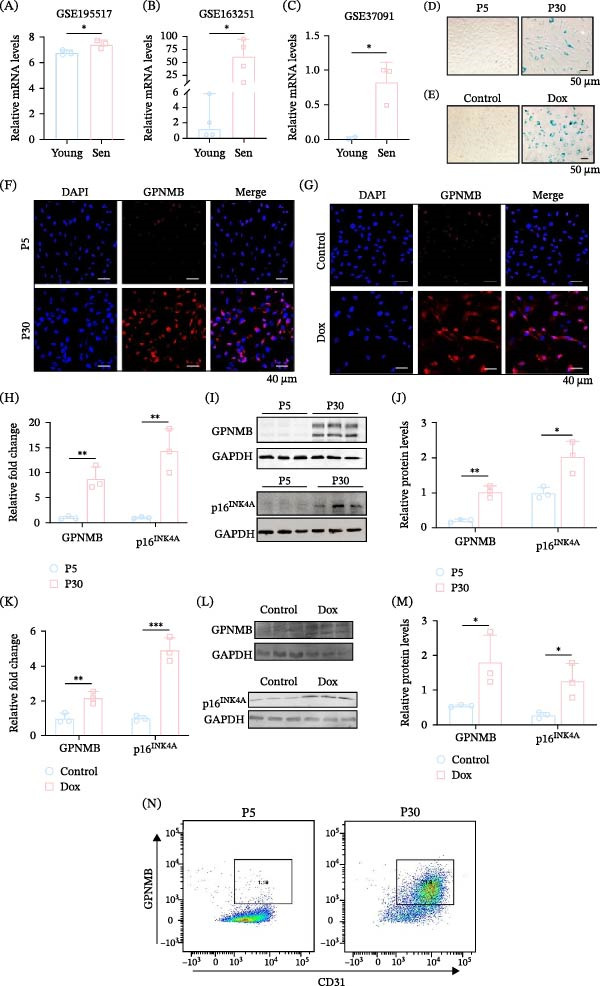
GPNMB expression is elevated in senescent HUVECs. (A–C) Relative mRNA levels of GPNMB in senescent HUVECs from the Gene Expression Omnibus database. (D, E). SA‐β‐Gal activity in HUVECs undergoing replicative senescence or Dox‐induced senescence. Scale bars, 50 μm. (F, G). Immunofluorescence staining for GPNMB expression in HUVECs undergoing replicative senescence or Dox‐induced senescence. Nuclei are stained with DAPI (blue). Scale bars, 40 μm. (H–J). Relative mRNA fold change and western blot analysis of GPNMB and p16^INK4A^ in HUVECs undergoing replicative senescence. (K–M) Relative mRNA fold change and western blot analysis of GPNMB and p16^INK4A^ in HUVECs undergoing Dox‐induced senescence. (N) GPNMB surface expression of young and senescent HUVECs was analyzed by flow cytometry. Three independent experiments were performed. Data represent the mean ± SD. Data were compared by unpaired Student’s *t* test.  ^∗^ *p* < 0.05;  ^∗∗^ *p* < 0.01;  ^∗∗∗^ *p* < 0.001.

To explore the expression level of GPNMB in senescent ECs, we first analyzed three gene expression profiles (GSE195517, GSE163251, and GSE37091) obtained from the Gene Expression Omnibus database. The GPNMB mRNA expression level was higher in the senescent HUVECs than in the young group (Figure 1A–C). Next, we constructed two EC senescence models, replicative senescence and Dox‐induced senescence. A flattened morphology and enhanced SA‐β‐Gal activity were observed in these two models (Figure 1D,E). Moreover, increased GPNMB protein expression was also detected in senescent ECs (Figure 1F,G). The mRNA and protein expression of GPNMB and p16^INK4A^ were increased significantly in these senescent cells (Figure 1H–M, Supporting Information 3: Figures S1–S2). We observed an increased GPNMB surface expression in HUVECs using flow cytometry (Figure 1N). Overall, GPNMB expression was increased in senescence HUVECs, and these results support the notion that GPNMB could be a molecular target for senolytic therapy.

### 3.2. GPNMB‐Targeted CAR‐T Cell

**Figure 2:**
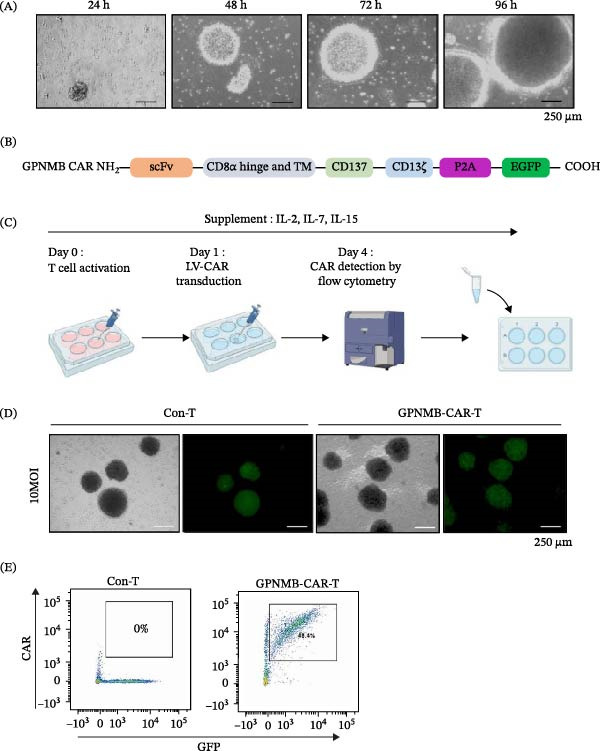
The construction of targeting senescent cells with GPNMB‐CAR‐T cells. (A) T cells from human peripheral blood lymphocytes culture and expansion. Scale bars, 250 μm. (B) CAR structure. (C) The production procedure of CAR‐T cells. (D) GFP detection in T cells at day 3 after CAR transduction. Scale bars, 250 μm. (E) Increased GPNMB scFv expression in GPNMB‐CAR‐T cells was detected by flow cytometry.

To generate GPNMB‐targeted CAR‐T cells, we first isolated human T cells from the PBMCs of healthy donors. We observed the activation and expansion of T cells treated with CD3/CD28 T cell activator, followed by hIL‐2, hIL‐7, and hIL‐15 for 96 h. It is obvious that the number of T cells increases with the increasing culture time (Figure 2A). Next, we constructed a second‐generation CAR, comprising the GPNMB scFv, CD8α hinge and transmembrane region, CD137 (4‐1BB) costimulatory domain, and CD3ζ intracellular signaling domains, and cloned them in frame into a lentivirus vector (Figure 2B). The lentiviral CAR vector was transduced into human T cells on day 1 after activation. GFP expression was detected in successfully transfected cells (CAR‐T cells) with a fluorescence microscope (Figure 2C,D). Moreover, using flow cytometry, we observed increased GPNMB scFv expression in GPNMB‐CAR‐T cells (Figure 2E). Taken together, these results demonstrated that GPNMB‐targeted CAR‐T cells were successfully constructed.

### 3.3. GPNMB‐CAR‐T Cells Selectively Eliminated Senescent HUVECs

**Figure 3:**
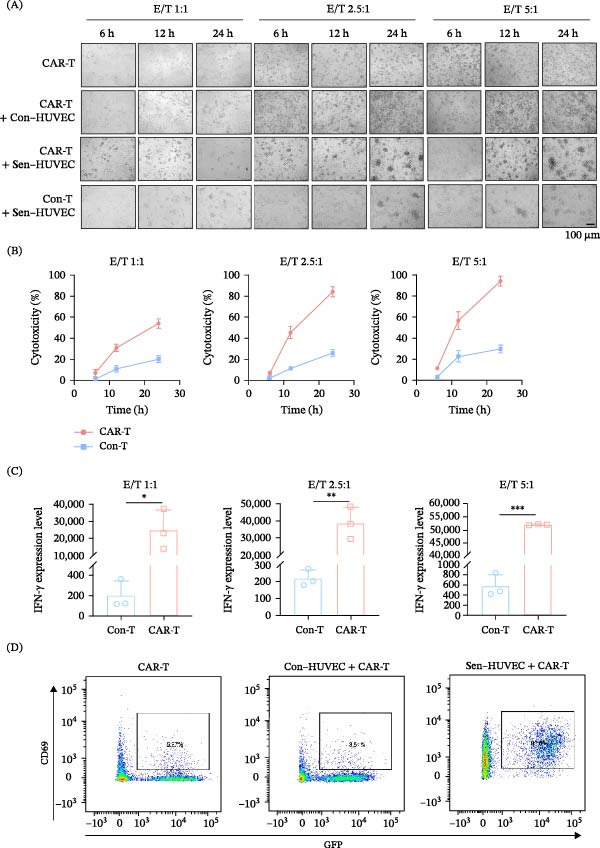
GPNMB‐CAR‐T cells selectively eliminate senescent HUVECs. (A, B) Cytotoxicity of Con‐T and GPNMB‐CAR‐T (CAR‐T) cells to replicative senescence HUVECs at an E:T ratio of 1:1, 2.5:1, and 5:1 after 6, 12, 24 h of coculture. The maximum LDH release control was obtained by lysing target cells with 10% Triton X‐100 for 20 min prior to supernatant collection. Data are representative of *n* = 3. Scale bars, 100 μm. (C) The concentrations of cytokines IFN‐γ at 24 h of coculture was quantified in the coculture supernatant. (D) CAR‐T cells were stimulated with young or senescent HUVECs for 24 h and CD69 expression was analyzed by flow cytometry. Three independent experiments were performed. Data represent the mean ± SD. Data were compared by unpaired Student’s *t* test.  ^∗^ *p* < 0.05;  ^∗∗^ *p* < 0.01;  ^∗∗∗^ *p* < 0.001.

Further studies explored the killing activity of GPNMB‐CAR‐T cells using replicative senescence of HUVECs in vitro. Here, we performed killing assays coculturing senescent HUVECs and GPNMB‐CAR‐T cells at different E:T ratios and evaluated killing at 6, 12, and 24 h. As we expected, the enhanced killing effects of GPNMB‐CAR‐T cells were observed as the E/T ration increased compared to the control group. Moreover, GPNMB‐CAR‐T cells efficiently killed GPNMB‐expressing HUVECs but not young HUVECs (Figure 3A). GPNMB‐CAR‐T cells efficiently eliminated senescent HUVECs (Figure 3B) and produced increased concentrations of IFN‐γ (Figure 3C). Additionally, after coculture with senescent HUVECs, GPNMB‐CAR‐T cells showed a notable upregulation of CD69, an indicator of T‐cell activation (Figure 3D). In summary, the targeting of GPNMB with CAR‐T cells appears to selectively and effectively eliminate senescent ECs.

### 3.4. GPNMB‐CAR‐T Cells Consume Senescent HUVECs in Matrigel Mimicking the In Vivo Environment

**Figure 4:**
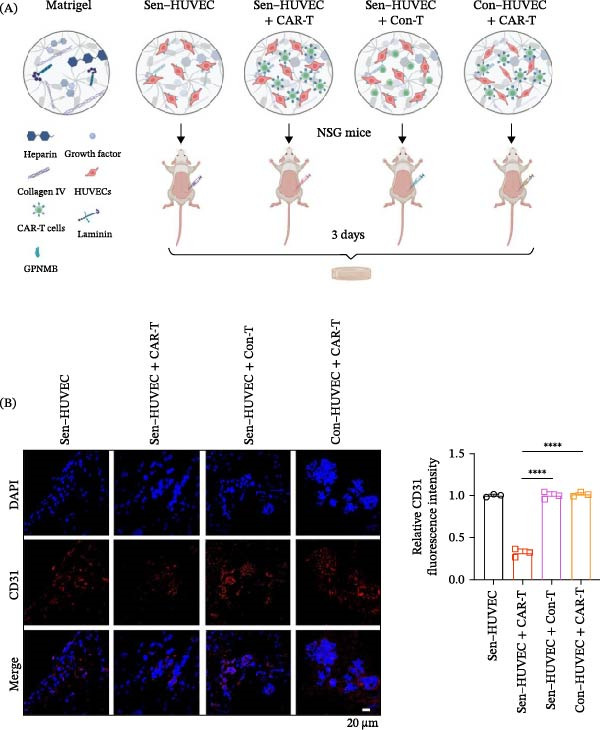
GPNMB‐CAR‐T cells consume senescent HUVECs in Matrigel mimicking the in vivo environment. (A) The diagram showing the cytotoxicity assay procedure of CAR‐T cells in Matrigel. (B) Immunofluorescence staining for CD31 expression and quantification in Matrigel plugs. Scale bars, 20 μm. Three independent experiments were performed. Data represent the mean ± SD. Data were compared by unpaired Student’s *t* test.  ^∗∗∗∗^ *p* < 0.0001.

To investigate the cytotoxic activity of GPNMB‐CAR‐T cells against senescent HUVECs in vivo condition, we mixed CAR‐T cells and young or senescent HUVECs in the Matrigel, which were then implanted subcutaneously into immunodeficient mice (Figure 4A). Three days after implantation, the Matrigel plugs were isolated from mice, and the density of remaining HUVECs was analyzed by staining CD31 (also known as PECAM‐1), a well‐established vascular EC marker. We found that, in contrast to Con‐CAR‐T cells, GPNMB‐CAR‐T cells successfully eliminated the senescent HUVECs but not young HUVECs. (Figure 4B).

## 4. Discussion

In this study, we showed that CAR‐T cells could eliminate senescent ECs by targeting GPNMB in vitro and in Matrigel plugs implanted into mice, providing a new senolytic strategy.

Accumulating evidence suggests that the selective removal of senescent cells can alleviate tissue dysfunction and delay age‐associated diseases [16]. Traditional senolytic strategies relying on small‐molecule compounds often lack specificity and may produce systemic toxicity, prompting the emergence of immune‑based alternatives such as CAR‐T cell‑mediated clearance. The success of senolytic CAR‐T therapies hinges on the precise identification of surface senescence‐associated antigens. For example, CAR‐T cells targeting natural killer group 2 member D ligands (NKG2DLs) or urokinase plasminogen activator receptor (uPAR) have shown efficacy in experimental models [17, 18]. However, NKG2D‑based CAR‑T cells broadly recognize stress‑induced ligands, raising the risk of off‑target cytotoxicity against nonsenescent but transiently stressed cells. Meanwhile, anti‑uPAR CAR‑T cells reduce liver senescence in aged or high‑fat‑diet mice [19], but their ability to target senescent aortic ECs requires further investigation. Consistent with these studies, our results suggest that GPNMB may represent another potential surface marker that can be exploited for senolytic immunotherapy.

Endothelial senescence is a key contributor to vascular aging and contributes to multiple cardiovascular and metabolic disorders. The targeted elimination of senescent ECs therefore represents a potential strategy to mitigate age‐related vascular dysfunction. Our findings identify GPNMB as a surface marker selectively upregulated in senescent ECs. Unlike NKG2D‐CAR‐T, which enables systemic senescent cell clearance, GPNMB‐CAR‐T may provide greater endothelial specificity tailored to vascular disease settings. Thus, these strategies may therefore be complementary rather than competitive depending on the disease context and therapeutic goals. Notably, two senolytic agents, quercetin and fisetin, currently in clinical trials, were initially recognized for their ability to selectively eliminate senescent ECs while sparing their nonsenescent counterparts [19, 20].

A recent work by Suda et al. [10] demonstrated that anti‐GPNMB vaccination can selectively eliminate senescent ECs through MHC class I‐restricted presentation of GPNMB‐derived peptides. This elegant strategy highlights the therapeutic potential of targeting GPNMB as a senescence‐associated antigen. In comparison, CAR‐T cells recognize the extracellular domain of target antigens in an MHC‐independent manner [21, 22], thereby circumventing potential limitations related to antigen processing efficiency, MHC polymorphism, or MHC downregulation under pathological conditions [23]. Vaccine‐induced immunity depends on endogenous immune competence and may be influenced by age‐associated immune decline [24, 25].

In contrast, CAR‐T cells provide immediate cytotoxic effector function upon antigen engagement and can undergo antigen‐driven expansion, potentially amplifying therapeutic efficacy beyond the initial infused dose [22, 26]. The dosing and treatment duration of CAR‐T cells can also be controlled with the incorporation of safety switches in engineered constructs [27, 28], which may be advantageous when rapid or adjustable senolytic intervention is required. Vaccination may be more suitable for long‐term prevention, whereas CAR‐T therapy could offer a more rapid and controllable option in vascular aging intervention.

Our study also showed several limitations. First, the current experiments primarily evaluated the short‐term cytotoxic activity of GPNMB‐CAR‐T cells. Future studies with longer observation periods will be necessary to determine the durability of senescent cell clearance and the persistence of CAR‐T cell activity. Second, although our data indicate selective targeting of senescent ECs, GPNMB has been reported to be expressed in multiple cell types under certain physiological or pathological conditions. In addition, GPNMB can be cleaved from the cell surface and exist as a soluble form, which may potentially influence CAR‐T cell engagement or contribute to off‐target effects. The safety and off‐target effects of GPNMB‐CAR‐T cell therapy should be evaluated in future studies. Finally, there are limitations in the in vivo study. The Matrigel plugs couldn’t fully mimic physiological or pathological vasculature. However, in this study, we used human T cells and the scFv of the antibody against human GPNMB. It is unknown whether this system could work well in mice. We need to test the targeting effects of GPNMB‐CAR to mouse GPNMB or perform an in vivo study in GPNMB‐humanized immunodeficient mice in the future.

## Author Contributions

Mei Zhou wrote the paper and performed experiments and data analysis. Peijie Yu performed experiments and data collection. Jie Du and Yan Liu supervised the study design and revised the paper.

## Funding

This study was supported by the National Natural Science Foundation of China (Grant 82300545).

## Ethics Statement

The use of human blood samples was approved by the Medical Ethics Committee of Beijing Anzhen Hospital, Capital Medical University, and was conducted in accordance with the principles of the Declaration of Helsinki. Informed consent was obtained from all participants.

## Conflicts of Interest

The authors declare no conflicts of interest.

## Supporting Information

Additional supporting information can be found online in the Supporting Information section.

## Supporting information


**Supporting Information 1** Table S1: The sequences of the vectors.

**Supporting Information 2** Table S2: The listed information of antibodies.

**Supporting Information 3** Figure S1: The original WB images of Figure 1I. Figure S2: The original WB images of Figure 1L. Figure S3: Representative gating strategy for flow cytometry in Figure 1N. The gating strategy for all plots was first to select live cells using FSC‐A/SSC‐A, and then to the identification of CD31⁺GPNMB⁺ senescent HUVECs. Figure S4: Representative gating strategy for flow cytometry in Figure 2E. The gating strategy for all plots was first to select live cells using FSC‐A/SSC‐A, and then to the identification of GFP⁺CAR⁺ T cells. Figure S5: Representative gating strategy for flow cytometry in Figure 3D. The gating strategy for all plots was first to select live cells using FSC‐A/SSC‐A, and then to the identification of GFP⁺CD69⁺ T cells.

## Data Availability

The data that support the findings of this study are available from the corresponding author upon reasonable request.
