# Supplementary material for: Targeted Clearance of Senescent Endothelium by GPNMB‐Specific CAR‐T Cells
Source: J Immunol Res. 2026 Aug 5;2026:1976386. doi: 10.1155/jimr/1976386 (PMC13439632; doi:10.1155/jimr/1976386)
Supplement: Supplementary file 1 [file JIMR-2026-1976386-s001.docx]

The listed information of antibodies

| *Antibodies* | *Source* | *catalog number* |
| --- | --- | --- |
| *Anti-P16 antibody* | *Abcam* | *Ab108349* |
| *Anti-GPNMB antibody* | *Cell Signaling Technology* | *13251* |
| *Anti-Human-GPNMB* | *invitrogen* | *2507003* |
| *Biotin anti-Human-CD31* | *invitrogen* | *2471649* |
| *FITC Conjugated Streptavidin* | *eBioscience* | *11-4317-87* |
| *Alexa Fluor 647 anti-His Tag Antibody* | *Biolegend* | *652513* |
| *APC anti-human CD69* | *BD Biosciences* | *560711* |
| *Anti-CD31 antibody* | *Servicebio* | *GB12064* |
| *Anti-GAPDH antibody* | *ZSGB-Bio* | *TA-08* |
| *β-actin* | *ZSGB-Bio* | *TA-09* |
