# Supplementary material for: Targeted Clearance of Senescent Endothelium by GPNMB‐Specific CAR‐T Cells
Source: J Immunol Res. 2026 Aug 5;2026:1976386. doi: 10.1155/jimr/1976386 (PMC13439632; doi:10.1155/jimr/1976386)
Supplement: Supplementary file 2 [file JIMR-2026-1976386-s002.docx]

**Supplementary Figure S1.** The original WB images of **Figure 1I**.

**Supplementary Figure S2.** The original WB images of **Figure 1L.**

**Supplementary Figure S3.** Representative gating strategy for flow cytometry in **Figure 1N**. The gating strategy for all plots was first to select live cells using FSC-A/SSC-A, and then to the identification of CD31⁺GPNMB⁺ senescent HUVECs.

**Supplementary Figure S4.** Representative gating strategy for flow cytometry in **Figure 2E**. The gating strategy for all plots was first to select live cells using FSC-A/SSC-A, and then to the identification of GFP⁺CAR⁺ T cells.

**Supplementary Figure S5**. Representative gating strategy for flow cytometry in **Figure 3D**. The gating strategy for all plots was first to select live cells using FSC-A/SSC-A, and then to the identification of GFP⁺CD69⁺ T cells.
