# Supplementary material for: Targeted Clearance of Senescent Endothelium by GPNMB‐Specific CAR‐T Cells
Source: J Immunol Res. 2026 Aug 5;2026:1976386. doi: 10.1155/jimr/1976386 (PMC13439632; doi:10.1155/jimr/1976386)
Supplement: Supplementary file 3 [file JIMR-2026-1976386-s003.docx]

**The sequences of the vectors**

CD8α hinge and TM:

accacgacgccagcgccgcgaccaccaacaccggcgcccaccatcgcgtcgcagcccctgtccctgcgcccagaggcgtgccggccagcggcggggggcgcagtgcacacgagggggctggacttcgcctgtgatatctacatctgggcgcccttggccgggacttgtggggtccttctcctgtcactggttatcaccctttactgc

CD137 (4-1BB) co-stimulatory domain:

aaacggggcagaaagaaactcctgtatatattcaaacaaccatttatgagaccagtacaaactactcaagaggaagatggctgtagctgccgatttccagaagaagaagaaggaggatgtgaactg

CD3ζ intracellular signaling domain:

agagtgaagttcagcaggagcgcagacgcccccgcgtacaagcagggccagaaccagctctataacgagctcaatctaggacgaagagaggagtacgatgttttggacaagagacgtggccgggaccctgagatggggggaaagccgagaaggaagaaccctcaggaaggcctgtacaatgaactgcagaaagataagatggcggaggcctacagtgagattgggatgaaaggcgagcgccggaggggcaaggggcacgatggcctttaccagggtctcagtacagccaccaaggacacctacgacgcccttcacatgcaggccctgccccctcgc
